# Enabling Psychiatrists to be Mobile Phone App Developers: Insights Into App Development Methodologies

**DOI:** 10.2196/mhealth.3425

**Published:** 2014-11-11

**Authors:** Melvyn WB Zhang, Tammy Tsang, Enquan Cheow, Cyrus SH Ho, Ng Beng Yeong, Roger CM Ho

**Affiliations:** ^1^National Healthcare GroupSingaporeSingapore; ^2^Institute of Mental HealthSingaporeSingapore; ^3^National University HealthCare SystemsSingaporeSingapore; ^4^Department of Psychiatry, Singapore General HospitalSingaporeSingapore

**Keywords:** smartphone application, mobile application, creation

## Abstract

**Background:**

The use of mobile phones, and specifically smartphones, in the last decade has become more and more prevalent. The latest mobile phones are equipped with comprehensive features that can be used in health care, such as providing rapid access to up-to-date evidence-based information, provision of instant communications, and improvements in organization. The estimated number of health care apps for mobile phones is increasing tremendously, but previous research has highlighted the lack of critical appraisal of new apps. This lack of appraisal of apps has largely been due to the lack of clinicians with technical knowledge of how to create an evidence-based app.

**Objective:**

We discuss two freely available methodologies for developing Web-based mobile phone apps: a website builder and an app builder. With these, users can program not just a Web-based app, but also integrate multimedia features within their app, without needing to know any programming language.

**Methods:**

We present techniques for creating a mobile Web-based app using two well-established online mobile app websites. We illustrate how to integrate text-based content within the app, as well as integration of interactive videos and rich site summary (RSS) feed information. We will also briefly discuss how to integrate a simple questionnaire survey into the mobile-based app. A questionnaire survey was administered to students to collate their perceptions towards the app.

**Results:**

These two methodologies for developing apps have been used to convert an online electronic psychiatry textbook into two Web-based mobile phone apps for medical students rotating through psychiatry in Singapore. Since the inception of our mobile Web-based app, a total of 21,991 unique users have used the mobile app and online portal provided by WordPress, and another 717 users have accessed the app via a Web-based link. The user perspective survey results (n=185) showed that a high proportion of students valued the textbook and objective structured clinical examination videos featured in the app. A high proportion of students concurred that a self-designed mobile phone app would be helpful for psychiatry education.

**Conclusions:**

These methodologies can enable busy clinicians to develop simple mobile Web-based apps for academic, educational, and research purposes, without any prior knowledge of programming. This will be beneficial for both clinicians and users at large, as there will then be more evidence-based mobile phone apps, or at least apps that have been appraised by a clinician.

## Introduction

Over the past decade, there have been massive developments in Web-based and Internet technologies, with the introduction of smartphones. Smartphones are a new generation of mobile phone technology that have created a revolution in the current telecommunications market [1]. Smartphones are currently equipped with immense computing capabilities that allow individuals to access the Internet on the go and have the capabilities to facilitate more than just the voice and text-based communications of cellular phones. In fact, they are generally now being regarded as handheld computers, rather than just cellular phones [1]. It was perhaps the release of Apple’s iPhone in 2007 that sparked a major revolution in the telecommunications and information technology arena. What was also regarded as pivotal in the advancement of mobile phone technology was the launch of the Apple App Store in July 2008 [2]. The app store enabled users to download mobile phone-based apps that allowed for additional capabilities on the phone, rather than their being used only as tools for accessing the Internet.

More recent studies have looked at medical students’ and trainees’ ownership, usage, and perspectives towards mobile phone use. In Payne’s 2012 study [2], a total of 257 medical students and 131 junior doctors were surveyed. The study showed a significantly high level of mobile phone ownership in that cohort. In addition, the majority of participants in their cohort owned between 1-5 medical-related apps. Compared to other platforms, iPhone users were more likely to own apps. Both the medical students and the trainee doctors had similar usage of apps, with most of them using apps for 20-30 minutes per day. The most frequently used apps include disease diagnosis, management, and drug reference apps. In a 2014 pilot study [3], Payne investigated the impact of implementation of a hospital-specific mobile phone app to a cohort of British junior doctors. The investigators created an iPhone app that contained mainly disease management and antibiotic dosing guidelines specific to a hospital and tested the app among 39 foundation year doctors for a total duration of 4 months. Their results showed that participants felt generally positive towards the availability of having such an app, with 68% indicating that the app helped them save significant time in clinical activities.

It is well known that anyone can publish a medical app and that the app stores do not routinely do a rigorous review of the accuracy of the app’s content prior to publication [4]. Given the fact that mobile phones are used by the majority of interns on a daily basis in performing their job, there is thus a need for more guidance and advice with regards to the information provided within each app to ensure that the information provided within is accurate and credible [5]. Previous studies [6-8] have demonstrated a lack of evidence base for apps for asthma self-management; prevention, detection, and management of cancer; and for cardiopulmonary resuscitation. If clinicians were more involved and took ownership of creating their own apps, there would be less concern about the evidence contained within apps. Recent studies have highlighted the need for clinicians to be more involved in the mobile phone app development process. A recent research article highlighted a simple methodology for creating an app using only an Internet browser and a text editor, but this does not eliminate the challenges faced by clinicians [9]. The methodology shared previously might help overcome the fears of clinicians who are keen to develop their own apps but lack technical skills. However, there may be still some resistance given the fact that some coding in computer programming language would still be required. In addition, there are limited features that could be integrated in the app based on the methodology that was shared previously.

The objective of the current research is to share two freely available methodologies for developing Web-based mobile phone apps, without needing to know any programming language. Furthermore, these methodologies allow not just programming of a Web-based app, but also integrating multimedia features within the app. The application and feasibility of these two methodologies are illustrated using an app developed by the authors.

## Methods

### WordPress

**Figure 1:**
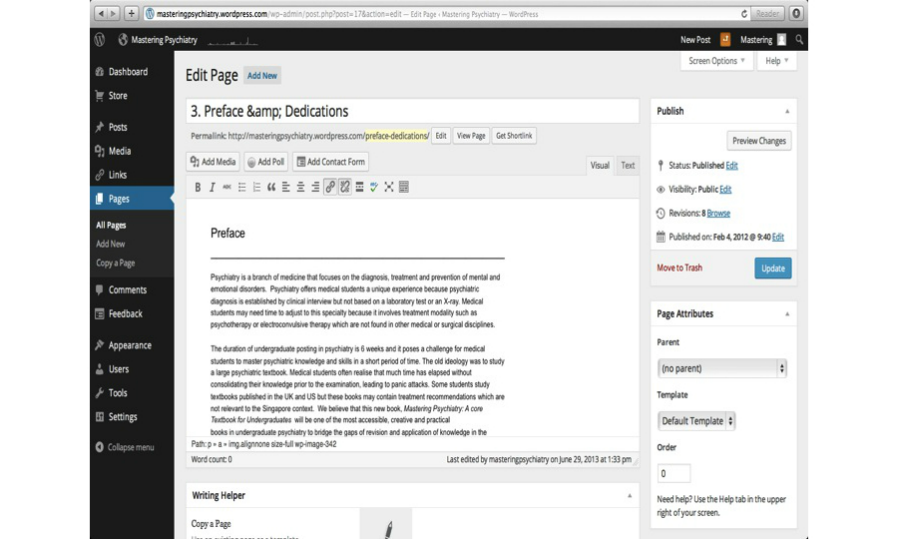
The graphic user interface of WordPress.

**Figure 2:**
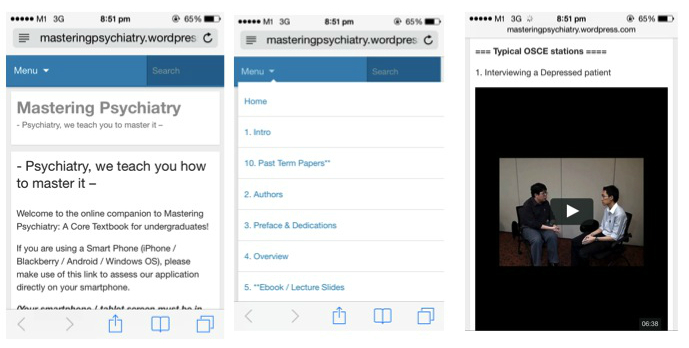
How WordPress automatically converts an online portal into a Web-based app.

There are currently two methodologies that can be used to create HTML5 mobile Web-based apps, without the need of any technical programming knowledge. The first option is using the WordPress portal [10], which has been commonly known to the general public as a blogging site. WordPress is online, open source website creation tool. When applied to medicine, all clinicians would need to do is to create an account, register for a domain name, and modify the content using the graphic user-interface that it offers (Figure 1). Text-based content integration is possible by dragging and dropping the appropriate content into the posts or pages. Multimedia features like videos can be uploaded to the library and automatically integrated into the page. The user can also generate forms, and filled forms can be directed to a clinician’s email address. When the portal is launched on a normal computer, it is a full-fledged website, but on a mobile phone, it is automatically displayed as an app (Figure 2).

### iBuildApp

The other methodology for developing a Web-based mobile app is by using online mobile Web-based app builders like iBuildApp [11]. Its graphic user interface would help in the immediate integration of text-based content, videos, and even rich site summary (RSS) feeds (for clinicians to retrieve information from a dedicated server). The authors have created a dedicated video to give clinicians an overview of the features (Multimedia Appendix 1) provided by current online app builders.

### Mastering Psychiatry App

The application of the above two methodologies has been illustrated through the development of the “Mastering Psychiatry” Web-based app. The Web-based portal and Web-based mobile phone app were developed between February and June 2012.

For the online portal (developed using WordPress), apart from the provision of a newly written textbook that integrates local clinical guidelines specific to Singapore, we included other features that could help augment student’s educational needs. These included videos demonstrating how to assess patients for a particular psychiatric disorder and how to elicit basic psychopathologies. In addition, an interactive multiple-choice survey was integrated that students could use for rapid revision of the multiple-choice component at their end for posting examinations.

For the Web-based app (developed using iBuildApp), we designed it to be a handy reference for students to use in their clinical settings. The app included the same content as was available on the online portal but was further subclassified into four individual tabs.

With regards to the deployment of the app, all of the students who were up for their clinical psychiatry posting were provided with information about the portal and the Web-based app on the first day of their clinical posting. A 10-minute demonstration of the features of both the online portal and the native app was conducted by the first author, MWBZ, on the first day of their clinical posting.

With ethics approval from the National University of Singapore, a user perspective survey was administered to the students right after completion of their end of posting clinical assessment. Participation in the survey was entirely voluntary, and a participant information handout was provided to all the participants prior to the start of the survey.

## Results

Our online portal and Web-based mobile phone app were launched on July 15, 2012, via direct dissemination of the Web links to the portal and to the mobile phone app. Since inception until today, there have been a cumulative total of 21,991 views of the online portal and a cumulative total of 722 users who have used the mobile app, based on our analytics. A cumulative total of 185 students took part voluntarily in the user perspective survey.

The majority of the students (141/178, 79.2%) students were 22 years old. More than half (121/227, 53.3%) used an Apple iOS device, whereas 21.6% (49/227) used an Android device. The majority of the students (124/186, 66.7%) had between 1-5 medical apps on their mobile phones. The purposes of the medical apps they had previously downloaded were mainly for educational purposes, as well as for usage in clinics and wards. The demographics of the sample population who took part in the survey are shown in Table 1.

The majority of the students (177/186, 95.2%) indicated that having a psychiatry mobile phone app would be useful. Similarly, a majority suggested that it would be particularly useful if the app contained textbook content and clinical videos. More than half of the students (105/184, 57.1%) agreed that having an app for psychiatry would be helpful, and 71.4% (132/185) also agreed that it would be a good companion to a traditional textbook. Table 2 gives an overview of students’ perspectives towards the app and its content.

**Table 1:** Student’s baseline demographic information.

Demographic variable		n	%
**Mobile phone ownership**			
	No	3	1.3
	iPhone	121	53.3
	Google Android	49	21.6
	iPad	24	10.6
	Android tablet	8	3.5
	Laptop / Notebook computer	22	9.7
**Medical-related app**			
	No	40	21.5
	1-5 apps	124	66.7
	6-10 apps	15	8.1
	11-15 apps	3	1.6
	>15	4	2.2
**Purpose of medical-related app**			
	Education – Revision	40	14.8
	Education – Learning	74	27.4
	Clinical (wards)	84	31.1
	Clinical (clinics)	55	20.4
	Others	17	6.3
**Gender**			
	Male	100	54.1
	Female	85	45.9
**Age in years**			
	20	1	0.6
	21	12	6.7
	22	141	79.2
	23	11	6.2
	24	12	6.7
	>25	1	0.6

**Table 2:** Student’s perspectives about app content and usefulness.

Perspectives		n	%
**Having mobile phone app for learning psychiatry**			
	Absolutely useless	2	1.1
	Useless	7	3.8
	Of some use	93	50.0
	Useful	60	32.3
	Very useful	24	12.9
**Usefulness of mobile phone app in psychiatry**			
	Absolutely useless	1	0.5
	Useless	5	2.7
	Of some use	73	39.7
	Useful	86	46.7
	Very useful	19	10.3
**Good companion to book**			
	Absolutely useless	4	2.2
	Useless	11	6.0
	Of some use	38	20.5
	Useful	94	50.8
	Very useful	38	20.5
**Having textbook contents in mobile phone apps**			
	Absolutely useless	4	2.2
	Useless	8	4.3
	Of some use	53	28.7
	Useful	84	45.4
	Very useful	36	19.5
**Having clinical OSCE videos in mobile phone apps**			
	Absolutely useless	2	1.1
	Useless	12	6.4
	Of some use	49	26.2
	Useful	81	43.3
	Very useful	43	23.0

## Discussion

### Principal Findings

The advantages of the website and app builder tools are that they enable clinicians to build their own apps that can be used for academic pursuits, education, and research. The initial findings in our study showed that a significant group of students were also amenable to trying out newer modalities of technologies, such as mobile phone technologies, to help them fulfill their educational needs on the go. The user perspective survey results showed that a high proportion of students valued the textbook and OSCE videos features in our app. A high proportion of students also agreed that a self-designed mobile phone app would be helpful for psychiatry education.

Due to the lack of previous research studies in this area, the interpretation of our results can be based only on a prior study on the development of a mobile phone app integrating guidelines for family physicians. Waldmann and Weckbecker [12] designed a Web-based app, very similar to ours in conception, that enabled teaching medical students more about primary care guidelines. In their Web-based app, a total of 15 guidelines from the German College of General Practitioners and Family Physicians (DEGAM) were included. Their study of 14 student testers showed that students preferred the Web-based app compared to a printed hard copy guideline. It was noted that students used the app much more frequently and made use of their waiting periods to go through the guidelines. They highlighted how the simple Web-based app helped to create an interest among students and helped them acquire valuable knowledge on the go or during down time. This might account for the initial results that we obtained, using the two methodologies that we described. Our students appeared to be similar to the cohort studied by Waldmann and Weckbecker [12], as they were receptive towards what we developed and perceived our Web-based self-developed psychiatry app as a useful tool for mastering psychiatry on the go. This reinforces the fact that our methodologies indeed do work and can equip psychiatrists with additional technological skills.

### Strengths and Limitations

The main strength of the current study is that we managed to assess the feasibility of enabling psychiatrists to be app developers and tested the student response to a self-developed app. Given that clinicians could regulate the content within the app if desired, and if there were collaborations between a group of clinicians, there would likely be some fundamental critical appraisal of app content. This is in line with Faye [13] who proposed that, in order to overcome the issue of the lack of apps that have been reviewed by appropriate authorities, we must demonstrate that the apps are evidence-based. One option involves having university or health care organizations creating their own in-house apps. It has been believed that developing in-house apps has inherent advantages, as the apps can help address specific shortfalls in clinical education, or specific deficiencies in competencies of either medical students or residents. Another suggestion would be for either university or health care organizations to have their own set of peer-reviewed apps. Other advantages of in-house apps are the low cost of development and the speed at which an app can be built. The speed of developing an app can be critical, especially if it has been planned for acquisition of information during a crisis. Finally, the advantages of using the website and app builders we described are that the apps created would be cross-compliant across various platforms and devices.

The limitations of the two methodologies we described include the fact that the number of interactive features that can be deployed are limited to what is in the app library online. Another limitation is that users would not be able to locate their app in the conventional app store, so dissemination of the availability of the app might be affected. Our sample size is relatively small and this is mainly due to the limited number of medical traineeship places available locally. In addition, our results are mainly derived from an Asian cohort and hence cannot be generalized to a Western cohort. We acknowledge that the response rate to our questionnaire is not 100%, as students might have skipped questions and not answered all the questions. In addition, we note that students might have given multiple responses to certain questions on a paper-based questionnaire, accounting for the varying total sample size for each question. It might be more ideal to consider allowing our students to fill in a Web-based online questionnaire with restricted responses to each question. We examined students’ perspectives from a sample that comprised mainly students with access to a smartphone. However, we do acknowledge that there are students with financial difficulties who might not have a smartphone, and hence our results might have an inherent bias.

### Conclusions

These Web and app-building methodologies can enable busy clinicians to develop simple mobile Web-based apps for academic, educational, and research purposes, without any prior knowledge of programming. This will be beneficial for both clinicians and users at large, as there will then be more evidence-based apps, or at least apps that have been appraised by a clinician. We hope that more work can be done in the future to address the limitations in the current study.

## Multimedia Appendix 1

Demonstration of Web-based mobile app with native app interface created by online application toolkits.

## Abbreviations

iOS: Apple operating system
OSCE: objective structured clinical examination
